# Emergency department visits for ambulatory care sensitive conditions by persons with Rheumatoid Arthritis: A population-based study

**DOI:** 10.1371/journal.pone.0337003

**Published:** 2025-12-10

**Authors:** Dani G. Contreras, Zanir Bhanji, J. Antonio Aviña-Zubieta, Claire E.H. Barber, Cheryl Barnabe

**Affiliations:** 1 McCaig Institute for Bone and Joint Health, University of Calgary, Calgary, Alberta, Canada; 2 Arthritis Research Canada, Vancouver, British Columbia, Canada; 3 Department of Kinesiology and Haskayne School of Business, University of Calgary, Calgary, Alberta, Canada; 4 University of British Columbia, Vancouver, British Columbia, Canada; 5 Departments of Medicine and Community Health Sciences, Cumming School of Medicine, University of Calgary, Calgary, Alberta, Canada; Virginia Commonwealth University School of Medicine, UNITED STATES OF AMERICA

## Abstract

**Purpose:**

We estimated emergency department (ED) visit rates for Canadian-indicator Ambulatory Care Sensitive Conditions (ACSCs) by persons with rheumatoid arthritis (RA) relative to age- and sex-matched general population controls.

**Methods:**

Cases were identified using a validated definition based on International Classification of Diseases codes (years 2002–2023). We identified visits in the National Ambulatory Care Reporting System (NACRS) where the most responsible diagnosis was for any ACSC (grand mal seizures, chronic lower respiratory diseases, asthma, diabetes, heart failure and pulmonary edema, hypertension, angina) and extracted visit acuity. Annual incidence rates were calculated within five years from the index date. The incidence rate ratio between RA and non-RA was estimated using a multivariable regression model, adjusting for age, sex, location of residence, and socioeconomic status.

**Results:**

RA (n = 35,770 individuals) had higher ED visit rates for all ACSCs combined compared to Non-RA (n = 94,094 individuals) (crude IRR 1.26, 95% CI 1.22, 1.31), persisting after adjusting for confounders (adjusted IRR 1.30, 95% CI 1.25, 1.34). More than two-thirds of ED visits for ACSCs were triaged as “urgent” or higher severity. Over the study period, there was a 34% increase in the proportion of ED visits for an ACSC condition among those with RA.

**Conclusion:**

RA cases had a 30% higher rate of avoidable ED visits in the first 5 years following diagnosis compared to non-RA. Improved ambulatory care access and care quality, inclusive of primary care and subspecialty care, is proposed to reduce the burden on the acute care system.

## Introduction

The consequences of systemic inflammation in Rheumatoid Arthritis (RA) are posited to lead to increased healthcare utilization, including emergency department (ED) visits, which drives healthcare system costs. In Alberta, persons with RA are overrepresented in ED utilization. In 2017, 2.1% of all the ED visits in the province were made by persons with RA [1] despite the prevalence of the condition only being 1.1% in Alberta [2]. Some of these ED visits may be avoidable.

Ambulatory Care Sensitive Conditions (ACSCs) are conditions where appropriate and timely access to ambulatory care could prevent complications, a more severe disease course, or the need for ED visits and hospitalizations, and are also used as indicators of primary care access [3]. Accepted ACSCs used for surveillance of the Canadian health care system performance include grand mal status and other epileptic convulsions, chronic lower respiratory diseases, asthma, diabetes, heart failure and pulmonary edema, hypertension, and angina [3]. People with RA remain at increased risk for some of these, including excess morbidity and mortality related to comorbid conditions such as cardiovascular [4,5] and chronic pulmonary diseases [6] compared to the general population.

Canada has one of the richest and most comprehensive collections of health data, including administrative health databases that record patient interaction with the healthcare system, including emergency and urgent care visits, hospitalizations, outpatient physician visits, and pharmacy dispensations. We conducted this study to investigate whether ED visits by RA patients are driven by conditions that could be avoidable. Our objective was to estimate the frequency of ACSC-related ED visits by persons with RA relative to the general population, characterize acuity at presentation, and examine whether there have been temporal changes in use over a 17-year time period.

## Materials and methods

Study Design: This was a retrospective cohort study using population-level administrative health data. In Alberta, a province of 4.6 million people, all health administrative data are maintained by Alberta Health for the Alberta Health Care Insurance Plan (AHCIP) and Alberta Health Services, as a single-payer health system. The administrative datasets represent patient interactions with the healthcare system at all sources (ambulatory care with primary care physicians and specialists; emergency department and urgent care visits; hospital admissions; pharmacy dispensations). Linkage for cohort creation and outcome ascertainment is made possible through a Unique Lifetime Identifier (ULI) assigned to each patient.

Datasets: For this project, four datasets were accessed: the Discharge Abstract Database (hospitalizations, April 2007-March 2023), Practitioner Claims (inpatient/outpatient physician visits, April 2002-March 2023), National Ambulatory Care Reporting System (emergency department and urgent care visits, April 2007-March 2023), and Provincial Registry (demographic information, April 2002-March 2023). The data was accessed on 16/04/2025.

Participants: A cohort of persons meeting a validated case definition for RA [7,8] was extracted from the datasets using an algorithm with diagnostic codes from the International Classification of Diseases Ninth Revision (ICD-9) for Practitioner Claims, or Tenth Revision (ICD-10) for Discharge Abstract Database for RA (in ICD-9-CM 714.X and ICD-10-CA M05.X-M06.X). Those included in the cohort must have had one hospitalization discharge or at least two practitioner claims for RA in two years, but at least 8 weeks apart (97% sensitivity, 77% specificity, 67% PPV, 98% NPV) [8]. There was a conservative five-year washout period applied to establish an incident cohort and exclude prevalent cases. To compare estimates to the general population not meeting the criteria for RA, 1:4 sex- and age-matching (within five years) was employed. The index date for RA cases was the date of first diagnostic code for RA, and this same date was applied as the index date for each case’s matched controls. The controls were randomly selected among all eligible persons enrolled in the AHCIP. We excluded individuals younger than 18 years of age, and those meeting case definitions of other inflammatory arthritis conditions of psoriatic arthritis (696.X, 720.X, L40.X, M07.0, M07.1, M07.2, M07.3 and M45.X), ankylosing spondylitis (720.X, M45.X), and gout (274.X, 712.X, M10.X, M11.X).

Outcomes: The primary outcome of interest was ED visit rates for any ACSC, and individual ACSCs, calculated as incidence rate ratios (IRR) (95% CI)) for RA relative to non-RA. Annual rates of visits were calculated for a 5-year period following the index date. We applied the defining ICD-10-CA/CM codes for each ACSC following the Canadian Institute for Health Information (CIHI) methodology (See S1 Table). The acuity of ACSC visits as recorded at presentation using the Canadian Triage and Acuity Scale (CTAS) (1 Resuscitation, 2 Emergent, 3 Urgent, 4 Less urgent, 5 Non-urgent) [9] was a secondary outcome.

Statistical analyses: Descriptive statistics were used to report the characteristics of ACSC ED visits up to five years from the index date for RA and non-RA. Missing values of location of residence (urban/rural) and socioeconomic status (determined using the Pampalon Deprivation Index [10]) were imputed using multiple imputation by chained equations [11]. IRRs (95% CI) for each ACSC were calculated using a zero-inflated Poisson regression model, adjusting for age at presentation (continuous), biological sex (male/female), location of residence determined using forward sortation area [12], and socioeconomic status. The model was checked to diagnose possible multicollinearity using Variance Inflation Factor [13]. Statistical analyses were performed using R version 4.4.0 packages *pscl* [14] for zero-inflated Poisson regression, and *car* [13] for model validation.

### Ethics

Ethics approval for this study was provided by the University of Calgary Conjoint Health Research Board (Ethics ID REB22–1316). All patient data were fully deidentified by the data custodians; therefore, informed consent was waived by the ethics committee.

## Results

Cohort and Visit Characteristics (Table 1).

**Table 1:** Characteristics of RA and Non-RA that had an ED visit for any reason during the study period.

	RA Cases (N=35,770 individuals with an ED visit)	Non-RA (N=94,094 individuals with an ED visit)
ED Visits	140,589	233,571
Mean number of ED visits within 5 years (SD)	5.97 (13.0)	3.75 (5.72)
Female sex	140,589 (65.79)	233,571 (66.22)
**Age at ED visit, years**		
Mean (SD)	58.01 (17.53)	65.42 (16.93)
Median (IQR)	58 (46, 71)	68 (54, 79)
**Rural Location of residence**	73,808 (34.54)	108,726 (30.83)
**Socioeconomic status**		
1 – Materially and socially privileged DA^a^	19,189 (8.98)	38,050 (10.79)
2 – DA with a tendency towards privilege	25,419 (11.90)	45,031 (12.77)
3 – DA privileged on one dimension but deprived on the other	43,622 (20.41)	62,367 (17.68)
4 – DA with a tendency towards deprivation	46,526 (21.77)	77,148 (21.87)
5 – Materially and socially deprived DA	78,926 (36.94)	130,117 (36.89)
**Ambulatory Care Sensitive Condition**		
Angina	674 (0.32)	1,661 (0.47)
Asthma	785 (0.37)	1,278 (0.36)
Chronic lower respiratory diseases (except asthma)	3,399 (1.59)	6,929 (1.96)
Diabetes	874 (0.41)	1,560 (0.44)
Grand mal status and other epileptic convulsions	333 (0.16)	481 (0.14)
Heart failure and pulmonary edema	1,775 (0.83)	4,232 (1.20)
Hypertension	1,330 (0.62)	3,949 (1.12)
No ACSC	204,512 (95.71)	332,623 (94.30)

^a^DA: Dissemination area; Combined social and material deprivation index from Pampalon quintiles [10]

The incident cohort consisted of 52,596 individuals with RA, matched to 210,384 randomly selected individuals who did not meet the RA case definition. From the RA cohort, 68% (n = 35,770 individuals) had at least one ED visit for any reason up to five years after their index date. This proportion is significantly higher compared to non-RA (n = 94,128 individuals, 44.7%). The average age at ED visit was 58 (SD 17.5) years for RA and 65.4 (SD 16.9) years for non-RA, with no difference in sex proportion (approximately 66% identified as female sex for both cohorts). Those with RA had an average of 6 (SD 13.0) visits per person throughout the 5-year observation study period, compared to 3.8 (SD 5.7) visits per person among non-RA (p < 0.001).

Approximately 4.3% (9,170 out of the total 140,589 ED visits) of all ED visits by RA cases had a most responsible diagnosis of an ACSC. Among individual ACSCs, ED visits for chronic lower respiratory diseases comprised more than 30% of all ACSC ED visits: 37% for all ACSC ED visits for RA and 34% for non-RA, respectively. This was followed by heart failure and pulmonary edema (19% for RA and 21% for non-RA), hypertension (15% for RA and 20% for non-RA), diabetes (9.5% for RA and 7.8% for non-RA), asthma (8.6% for RA and 6.4% for non-RA), angina (7.4% for RA and 8.3% for non-RA), and grand mal seizures (3.6% for RA and 2.4% for non-RA).

Incidence rate ratios (Table 2).

**Table 2:** Crude and adjusted incidence rate ratios (95% Confidence Interval) of ACSC ED visits.

Ambulatory Care Sensitive Condition	Unadjusted	Adjusted^b^
Overall	1.26 (1.22, 1.31)	1.30 (1.25, 1.34)
Grand Mal Seizures	1.32 (1.07, 1.62)	1.08 (0.87, 1.33)
Chronic lower respiratory diseases	1.15 (1.09, 1.21)	1.16 (1.10, 1.22)
Asthma	1.26 (1.09, 1.47)	1.19 (1.03, 1.38)
Diabetes	1.40 (1.22, 1.59)	1.12 (0.98, 1.28)
Heart failure and pulmonary edema	1.25 (1.15, 1.36)	1.26 (1.16, 1.38)
Hypertension	1.00 (0.88, 1.13)	1.09 (0.97, 1.23)
Angina	1.21 (0.97, 1.50)	1.21 (0.97, 1.52)

^b^Adjusted IRR – Incidence rate of RA: Non-RA patients. Adjusted by age, sex, geographic location, and socioeconomic status.

The crude IRR for any ACSC ED visit was higher in RA cases compared to non-RA (IRR 1.26, 95% CI 1.22, 1.31). After adjusting for potential confounders, we determined that RA cases had a 30% higher rate of ED visits for any ACSC compared to non-RA (IRR 1.30, 95% CI 1.25, 1.34). The ED visit incidence rates were higher in those with RA for almost all the ACSCs, persisting after adjusting for confounders, except for hypertension and angina. The crude incidence rate for diabetes visits was higher among RA compared to non-RA (IRR 1.40, 95% CI 1.22, 1.59) but was not statistically different after adjusting for confounders (IRR 1.12, 95% CI 0.98, 1.28). This was also similar for ED visits for grand mal seizures, with the higher rates of ED visits not persisting after adjusting for confounders.

Acuity of ACSC Visits and Hospitalizations (Table 3).

**Table 3:** Acuity of ACSC related ED visits (n, %).

CTAS^c^	RA (N = 9,170)	Non-RA (N = 20,090)	Difference (RA vs Non-RA)
1 Resuscitation	158 (1.7)	361 (1.8)	−0.0001 (−0.0040, 0.0025)
2 Emergent	2,687 (29)	5,624 (28)	0.0131 (0.0019, 0.0245)
3 Urgent	4,161 (45)	9,289 (46)	−0.0086 (−0.0209, 0.0037)
4 Less urgent	1,488 (16)	3,372 (17)	−0.0056 (−0.0147, 0.0036)
5 Non-urgent	389 (4.2)	862 (4.3)	−0.0005 (−0.0055, 0.0045)
9 Unknown	287 (3.1)	582 (2.9)	0.0023 (−0.0019, 0.0066)

^c^CTAS = Canadian Triage Acuity Scale

Using the CTAS scores, 75.7% of all ACSC ED visits by RA cases were triaged as CTAS 1–3 (Resuscitation, Emergent, and Urgent) (1.7%, 29%, and 45%, respectively). This was comparable to the 75.8% of all ACSC ED visits by non-RA triaged CTAS 1–3.

**Fig 1:**
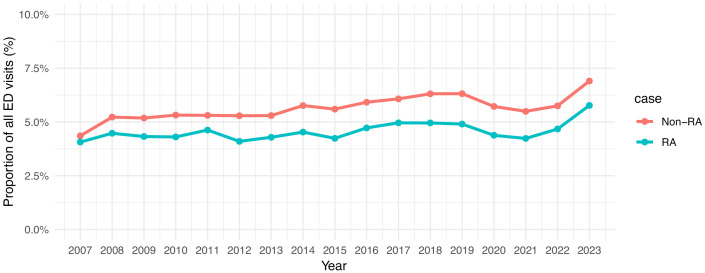
Proportion of ED visits for any Ambulatory Care Sensitive Condition (ACSC), relative to all ED visits, in RA and non-RA, by fiscal year end.

Over time, there was a 35% increase in the proportion of ED visits, as a proportion of all visits, that were for an ACSC among those with RA over the 17-year study period (Fig 1). In 2007, 4.1% of all ED visits by RA cases were for an ACSC, increased to 5.8% in 2023. In contrast, there was an approximate 45% increase in ACSC ED visits by non-RA over the same period, from 4.3% of visits in 2007 to 6.9% of all visits in 2023. Notable was a lower proportion of visits in both cohorts that were for an ACSC during the years 2020, 2021 and 2022, coinciding with the COVID-19 pandemic.

## Discussion

Our study contributes knowledge of ED utilization, and specifically ACSC-related healthcare utilization, by RA patients. Overall, individuals with RA more frequently attend the ED in the first five years following diagnosis as compared to their matched controls. Approximately 5% of these ED visits are potentially avoidable, and the proportion of visits for ACSCs has increased over the last decades. The risk of having an avoidable ED visit is elevated compared to those without RA. Over 75% of all ACSC visits were triaged “Urgent” or with higher severity, and approximately one-third resulted in hospital admission for both RA and non-RA. While our results are consistent with previous studies showing higher healthcare utilization by RA patients compared to those without RA [15,16], it demonstrates the increasing burden of avoidable health care use, and what proportion of these visits are amenable to intervention to reduce acute care use.

Close to 40% of these ACSC visits were for chronic lower respiratory diseases, which include emphysema and chronic obstructive pulmonary disease. It was not surprising that patients with RA present to the ED for cardiovascular and pulmonary conditions, as these are common complications and comorbidities of RA. A cross-sectional study across 17 countries examined the prevalence of comorbid conditions among RA patients and found a high prevalence of asthma (prevalence proportion 6.6%), cardiovascular events such as stroke and myocardial infarction (6%), and chronic obstructive pulmonary disease (3.5%) [17]. Comorbid conditions that are also ACSCs may increase healthcare utilization, particularly hospitalizations among RA patients [18].

This study also highlights that avoidable health care utilization estimates are amenable to intervention. There was a noticeable decline in ED visits during the period of the COVID-19 pandemic, likely driven by the increased social distancing restrictions resulting in fewer exacerbations of chronic lung disease, public avoidance of EDs and hospitals, and the attribution of COVID-related visits and admissions to the denominator of the estimates. Different approaches to seeking healthcare were likely employed. A study in Alberta examined patients with inflammatory arthritis (IA) conditions, which include RA, and found that 35% of the patients attempted to seek care from different providers prior to their ED visits (e.g., primary care provider, walk-in clinic, rheumatology clinic, virtual telehealth, the Health Link, an Alberta-based, nurse-led service providing health advice over the phone, or another healthcare provider such as a chiropractor, physiotherapist, or nurse practitioner) during a period coinciding with the pandemic [19].

While not all hospital admissions are avoidable, high-quality primary ambulatory care could mitigate the frequency or severity of presentations for ACSCs [3], and thereby reduce the need for ED visits and/or hospitalizations. Our results reinforce the need for collaborative care between ambulatory care physicians, inclusive of primary and specialty care, in the diagnosis, treatment, and management of RA and its comorbidities. In primary care settings, interprofessional teams have been shown to reduce avoidable hospitalizations, specifically for congestive heart failure [20]. Team-based care, which provides services by an interprofessional team, can ensure that patients can access health services in a timely and efficient manner. The Canadian Medical Association (CMA) highlighted the need to include not only physicians, but also allied health professionals, including psychologists, psychotherapists, and a registered dietitian, into a “primary care team” to improve access and quality of care [21]. Primary Care Networks in Alberta, Family Health Teams in Ontario, and Family Medicine Groups in Quebec all adopt a team-based primary care [21], although these structures are not available to every individual patient. Benefits of team-based care include a reduction in acute care service use; in Ontario, patients who were part of team-based care had slower increase in ED visits compared to patients in non-team-based care [22]. With the recent crisis in primary care access, 5.4 million Canadians 18 and older with no regular health provider [23], led to an influx of ED use for otherwise avoidable conditions. According to CIHI, approximately 15% of all ED visits between April 2023 to March 2024 could have been managed in primary care settings [24].

We acknowledge limitations of our study. Data in administrative health databases are collected in the operation of health services, not specifically designed for research and surveillance purposes. The diagnostic criteria applied to identify RA cases, while validated, may not fully include all persons with RA, and could include persons who do not have the condition too. We chose a widely used case definition that has been used by the Canadian Chronic Disease Surveillance System with optimal sensitivity and specificity (97% sensitivity, 77% specificity). There are also additional confounders, such as comorbidities, disease severity, treatment, smoking behaviour, and obesity, that were not accounted for as we did not have laboratory and diagnostic imaging data to estimate disease activity. Previous studies have noted that the definition of ACSCs only includes hospitalizations, but not ED visits. Therefore, a proportion of ED visits that do not lead to hospital admissions were not included in the estimates [25]. Finally, due to the nature of administrative data and lack of data availability, we also were not able to adjust for ethnicity. Despite these limitations, our research still provides data critical to understanding acute care use needs of persons with RA, so that a response through health system innovation can be developed and implemented.

## Conclusion

Persons with RA are at a higher risk of avoidable ED visits 5 years after diagnosis, with ED use for ACSC higher than the control population, and increasing over time. Better access to and quality of ambulatory care, including primary and specialty care, is necessary to reduce the burden on the acute care system and improve the quality of care for people with RA.

## Supporting information

S1 Table ICD codes for ACSCs as defined by the Canadian Institute for Health Information. (DOCX)
