# Supplementary material for: Emergency department visits for ambulatory care sensitive conditions by persons with Rheumatoid Arthritis: A population-based study
Source: PLoS One. 2025 Dec 10;20(12):e0337003. doi: 10.1371/journal.pone.0337003 (PMC12694863; doi:10.1371/journal.pone.0337003)
Supplement: S1 Table — (DOCX) [file pone.0337003.s001.docx]

S1 Table. ICD codes for ACSCs as defined by the Canadian Institute for Health Information.

| **Ambulatory Care Sensitive Conditions** | **Diagnostic codes** |
| --- | --- |
| Grand mal status and other epileptic convulsions | ICD-9/9-CM: 345  ICD-10-CA: G40, G41 |
| Chronic lower respiratory diseases (except asthma) | ICD-9/9-CM: 491, 492, 494, 496  ICD-10-CA: J41, J42, J43, J44, J47  **OR** MRDx^1^ of acute lower respiratory infection, only when a secondary diagnosis^2^ of J44 in  ICD-10-CA or 496 in ICD-9/9-CM is also present  ICD-9/9-CM: 466, 480–486, 487.0  ICD-10-CA: J10.0, J11.0, J12–J16, J18, J20, J21, J22 |
| Asthma | ICD-9/9-CM: 493  ICD-10-CA: J45 |
| Diabetes | ICD-9: 250.0, 250.1, 250.2, 250.7  ICD-9-CM: 250.0, 250.1, 250.2, 250.8  ICD-10-CA: E10.0, E10.1, E10.63, E10.64, E10.9, E11.0, E11.1, E11.63, E11.64, E11.9, E13.0, E13.1, E13.63, E13.64, E13.9, E14.0, E14.1, E14.63, E14.64, E14.9 |
| Heart failure and pulmonary edema^3^ | ICD-9/9-CM: 428, 518.4  ICD-10-CA: J81 (MRDx), I50 (MRDx), I50 as diagnosis type (1) when I11 is MRDx |
| Hypertension^3^ | ICD-9/9-CM: 401.0, 401.9, 402.0, 402.1, 402.9  ICD-10-CA: I10 (MRDx), I11 as MRDx when I50 as diagnosis type (1) is not present |
| Angina^3^ | ICD-9: 411, 413  ICD-9-CM: 411.1, 411.8, 413  ICD-10-CA: I20, I23.82, I24.0, I24.8, I24.9 |
| Diagnostic codes of cardiac procedure for exclusion | CCP: 47XX, 480X–483X, 489.1, 489.9, 492X–495X, 497X, 498X  ICD-9-CM: 336, 35XX, 36XX, 373X, 375X, 377X, 378X, 379.4–379.8  CCI codes beginning with: 1HA58, 1HA80, 1HA87, 1HB53, 1HB54, 1HB55, 1HB87, 1HD53, 1HD54, 1HD55, 1HH59, 1HH71, 1HJ76, 1HJ82, 1HM57, 1HM78, 1HM80, 1HN71, 1HN80, 1HN87, 1HP76, 1HP78, 1HP80, 1HP82, 1HP83, 1HP87, 1HR71, 1HR80, 1HR84, 1HR87, 1HS80, 1HS90, 1HT80, 1HT89, 1HT90, 1HU80, 1HU90, 1HV80, 1HV90, 1HW78, 1HW79, 1HX71, 1HX78, 1HX79, 1HX80, 1HX83, 1HX86, 1HX87, 1HY85, 1HZ53, 1HZ54, 1HZ55, 1HZ56, 1HZ57, 1HZ59, 1HZ80, 1HZ85, 1HZ87, 1IF83, 1IJ50, 1IJ55, 1IJ57, 1IJ76, 1IJ80, 1IK57, 1IK80, 1IK87, 1IN84, 1LA84, 1LC84, 1LD84, 1IJ86 **AND** not equal to (1HZ53LAKP, 1HZ55LAKP) **AND** not equal to abandoned at onset |

^1^MRDx = Most responsible diagnosis

^2^Secondary diagnosis = diagnosis other than MRDx

^3^Excluding cases with cardiac procedures. Exclusionary codes are listed.
